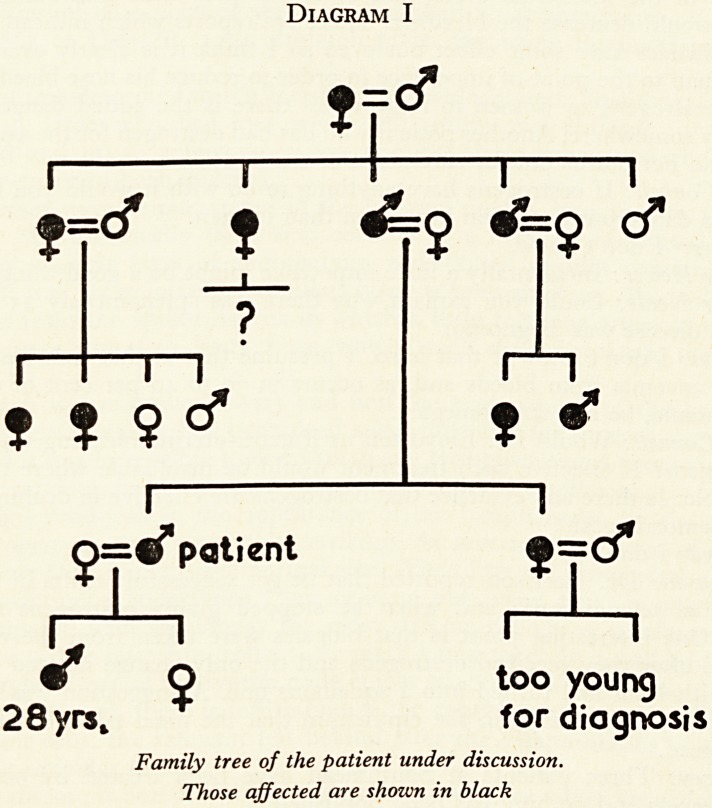# Hereditary Telangiectasia: Hodgkin's Disease

**Published:** 1958-10

**Authors:** T. F. Hewer


### HEREDITARY TELANGIECTASIA: HODGKIN'S DISEASE

(*A Clinical Pathological Conference of the University of Bristol Medical School*)

CHAIRMAN: PROFESSOR T. F. HEWER

*Dr. Cates:* This man, who was employed as a fireman in an aircraft factory, died from vomiting blood on the 7th December last year. He was a known victim of familial telangiectasia and had had several previous episodes of haemorrhage from the gut which had been very severe. Before he died he developed a fresh disease for which he received a course of nitrogen mustard.

In 1930, at the age of 31, he had a severe nose bleed, losing about one pint. He went to the General Hospital where his condition was recognized and he attended there on and off for the next twenty years, with bleeding occurring up to twenty times a year.

In 1932 he developed red spots on his face, nose and lips and then began to bleed from his tongue which was cauterized, and this stopped the bleeding. He was given a course of deep X-ray to the spleen (a treatment that would not nowadays be used for this condition). Nose bleeds continued for the next twenty years, otherwise he led a normal life.

In 1952 his haemorrhages became worse and he was subjected to a course of oestrogen therapy. It had been noticed that women with telangiectasia bleed more readily in the second two weeks of the menstrual cycle when oestrogen is not in the ascendant but progesterone is, so they thought it would be worth keeping him permanently under the effect of oestrogen. The effect on him of the intensive course was that he became impotent, his breasts became sore and swollen, he said he felt "awful" and that his nose was no better but bled just as much.

About 1956 he was referred to Mr. Pocock because he was having melaena. This was the first haemorrhage from the gut, the previous bleeds were all from the nose and mouth. As he also had indigestion the problem was whether this was bleeding from telangiectasia in his gut or had he two diseases. Did dyspepsia indicate an ulcer?

He was admitted and underwent gastroscopy. Mr. Capper reported that to every square inch of stomach lining there were three telangiectases and since there was no ulcer the bleeding must come from them. He was treated with iron and blood transfusion and was readmitted with a severe haemorrhage of the same sort in January and June 1957. He was also admitted to Frenchay Hospital as an emergency in the summer of 1957 with a severe nose bleed again.

About this time we wondered whether he should be subjected to a rather heroic form of surgery in which, Mr. Capper says, the stomach can be opened up and bleeding points cauterized; but before this was decided something else happened—though I'll come back to that later.

In December he had a profuse haemorrhage and was readmitted as an emergency and had altogether 13 pints of blood. At this time he had haematological changes which may be related to the treatment I am going to describe in a minute. During haemorrhages he became severely shocked and blood pressure went right down and stayed down. There was difficulty in matching blood because of his second disease and before he died his non-protein nitrogen rose to high levels and we thought his kidneys might be suffering as the result of an incompatible blood transfusion or of shock between transfusions. We decided that we were overtreating a man with another disease and put him on heroin and morphia; he died five days after admission.

Now, returning to the second disease, in September last year he had had 'flu, or what the layman calls 'flu, i.e. fever of unknown origin, headache, sweating, pains in limbs and weakness. In October he noticed swelling in the left side of the neck followed by swelling in his axillae. He was seen by Dr. A. D. M. Smith in the Outpatient Clinic where he was found to have a high temperature, and a large liver and spleen. It seemed likely that he had an acute reticulosarcoma. A biopsy was carried out and in view of the histological changes, which Dr. Johnson will describe later, it was decided to treat with nitrogen mustard. Two injections of 6 mgm of nitrogen mustard were given intravenously (the full course is six injections of 6 mgm) and his response was dramatic. His temperature dropped to normal and stayed normal, the patient himself felt miraculously better after the injections and the lumps themselves melted away completely after these injections. At this time it was noticed that he had a few petechiae and we were watching him for haematological changes as a result of this therapy. His W.B.C. was 2,000, platelets 40,000, at which level it was thought wisest to give no further treatment. Nitrogen mustard is known to be a dangerous substance, particularly from a haematological point of view, but when you have a patient critically ill with a malignant disease like this, the opinion is that it is better to make him feel better for the time being. His platelets went down to 40,000 and that perhaps contributed to the difficulty in controlling his haemorrhage when he came in a month later and died from the bleeding.

DIAGRAM I

*Family tree of the patient under discussion.*
*Those affected are shown in black*

The family history: This condition is transmitted as a Mendelian dominant. You will see from the chart (Diagram I) that one of his children, aged 28, also has telangiectasia. The patient inherited it from his father, and three of the father's brothers and sisters had it; in fact only one of them did not. Of his cousins three out of four in one family and both members in another family had it. There are other descendants from the patient's sister who are still young, and, as you probably know, the condition is not clinically obvious until the age of 35 to 45 or upwards, which makes it very difficult to prevent the transmission of the disease by eugenics.

*Dr. Johnson:* The histological appearance of the gland removed at biopsy showed complete disorganization of architecture with loss of lymph follicles and cellular invasion of the capsule. There was an increase in reticulin throughout the gland but little fibrosis. Cytological examination showed replacement of the normal elements with two types of cells, giant cells and reticulum cells together with a uniform admixture of lymphocytes.

This appearance is seen when Hodgkin's disease is advancing unusually rapidly and has been given the name Hodgkin's sarcoma.

*Professor Hewer:* This shows a much more malignant appearance than the usual run of Hodgkin's although there is every sort of gradation between this and the classical appearance. The term "Hodgkin's sarcoma" is not a very exact one. It is just an extremely active variant of the disease.

*Dr. Lloyd:* Could Dr. Cates give an explanation as to why oestrogen therapy is thought to influence bleeding from the telangiectases.

*Dr. Cates:* I feel this therapy is probably erroneous. The observation was that in premenopausal women epistaxis in this condition is sometimes worse in the second two weeks of the menstrual cycle, and so it was presumed that administration of oestrogen would decrease the bleeding. There are reports which indicate that oestrogens may in fact have some effect but even so I think it is clearly over-enthusiasm to treat a man to the point of impotence in order to reduce his nose bleeds; and when you give oestrogens to women in large doses there is the added danger of causing malignancy somewhere. Another patient who has had oestrogen for the same condition recently had her uterus out for carcinoma of the body.

*Dr. McConnell:* If oestrogens have anything to do with it would you not expect it to be a less dangerous condition in women than in men?

*Dr. Cates:* I don't know.

*Professor Hewer:* Incidentally a little impotence might be a good thing.

*Professor Neale:* Could you explain why there was splenomegaly 25 years before Hodgkin's disease was diagnosed?

*Dr. Cates:* I don't know if that is so. I presume that at that time he had an iron deficiency anaemia from bleeds and, as occurs in 30 to 40 per cent of cases of that sort of anaemia, he had splenomegaly.

*Dr. McConnell:* Would Dr. Lloyd tell us if extra-uterine bleeding was affected by the oestrogens? If effective such treatment would be invaluable where transfusion is not available. Is there any evidence that oestrogens are effective in dealing with extra-uterine haemorrhages?

*Dr. Lloyd:* I don't know.

*Dr. Johnson:* Dr. Harrison reported that he got successful results in four patients with familial telangiectasia and when he stopped giving oestrogens the bleeding recurred. One interesting point is that biopsies were taken from the nasal mucosa before and after they received oestrogens and the only change noticed was that the normal epithelium had turned into a squamous one. A suggestion was made that it was because of this change in the eipthelium that the nasal telangiectases were less likely to bleed.

*Dr. Tovey:* Three patients at Southmead have been treated by oestrogens but without success and nothing has been published.

*Professor Neale:* This seems to be a case of history repeating itself, twenty to twenty-five years ago uterine extracts were recommended for haemophilia.

*Dr. Cates:* The trouble is that negative findings are usually left unsung; if this work were published it would save other people from duplicating the experiments to get the same negative result.

*Professor Neale:* Do we know the age range of the members of this family who are not affected?

*Dr. Cates:* No.

*Professor Neal:* Was there any evidence of retinal telangiectasia?

*Dr. Cates:* I don't know.

*Professor Neale:* Isn't this a disease in which no retinal changes are found?

*Dr. Cates:* Yes.

*Dr. Spence:* If he had not developed disease No. 2 what was his likelihood of living a normal life?

*Dr. Cates:* These people do die of haemorrhage when they have developed about as widespread telangiectases as he had, they have big haemorrhages needing blood transfusions one after another.

*Dr. Tovey:* The oldest I know is a woman of sixty-five who has now reached the stage of transfusions every fortnight.

*Dr. Johnson:* At post-mortem this man had about a dozen telangiectases palely outlined on the lips. Straw coloured fluid was present in the abdominal cavity, the peritoneal surface was slightly blood-stained and there were blood-stained effusions in the pleural cavities. The lungs showed a little patchy polymorph infiltration. The chambers of the heart were normal but on the cusps of the mitral valve there were some so-called marantic vegetations (Interruption: Not endometrial tissues from the oestrogens?), made up of organizing fibrin, an incidental finding of little importance.

The condition of telangiectasia was first described by Babbington in 1865 (not by Rendu in 1868 or Osler in 1904 although given the name Rendu-Osler disease!) (Laughter).

One family tree described in the literature particularly beats this case; it is that of a Mormon in Utah who had four wives and twenty-two children, of whom ten were affected and up to a few years ago amongst the descendants who had been traced eighty-two had been affected.

Telangiectases were demonstrated in the internal organs, best seen in the capsule of the liver. Microscopically the lesion consists of a network of dilated capillaries lined only by a single layer of endothelium and dilated venules containing a little elastic in their wall. It is, in fact, a capillary-venous haemangioma. You will note that it is different from the spider naevus in which a little tortuous arteriole rises in the centre to form a pulsating body from which small dilated vessels, the legs, run out.

The stomach was examined to try and find the exact source of the haemorrhage but unfortunately this man had been dead some time and the mucosa was so heavily blood-stained it was impossible to distinguish any telangiectases. A section of stomach wall taken at random, however, showed numerous dilated capillaries.

The changes produced in the appearance of the lymph nodes by the two recent doses of nitrogen mustard were quite striking. At post-mortem none of the lymph nodes was more than about twice normal size. When first cut the surface presented a translucent gelatinous appearance from oedema but when fixed it was white and opaque. Compared with the biopsy the most striking change is an almost complete disappearance of the reticulum cells, their place being taken by small adult lymphocytes. The architecture of the lymph node is just as abnormal, however; the reticulin has become condensed, there is infiltration of the capsule and one can still make out abnormal giant cells, the remnant left behind after the disappearance of so much of the malignant reticular tissue.

Hodgkin's disease is one of the few malignant reticuloses which react fairly well to irradiation or treatment with cyto-toxic drugs. The cytological changes demonstrated in this case are similar to those which would have been seen after irradiation but take a little longer to appear.

The spleen was enlarged, congested and contained a large number of lymphocytes and a few abnormal reticulum cells.

The kidneys were swollen but showed no changes other than interstitial oedema, There was no tubular necrosis.

The femur showed extension of the red marrow throughout the upper two thirds of the shaft. This is a reactive hyperplasia in response to blood loss and possibly, but we have no proof, to a haemolytic reaction to the Hodgkin's disease.

*Question:* There is no evidence, is there, that Hodgkin's is produced by irradiation as are the leukaemias?

*Dr. Johnson:* I don't think so.

*Professor Hewer:* Would anybody like to tell us about the cytotoxic action of nitrogen mustard?

*Dr. Johnson:* I don't think anyone really knows. Irradiation produces ionization in the cells, so it is said. Nitrogen mustard probably interferes with cell metabolism in some other way but the end result is the same.

*Student:* What is the difference between a spider naevus and a telangiectasis?

*Dr. Cates:* The difference at the bedside is that a spider naevus has an arteriole in the centre and a leash of dilated capillaries coming from that and if the arteriole is obliterated by pressure with a sharp instrument all the legs of the spider disappear at once. They are mainly seen on the face in ordinary people but particularly in pregnant women and in cases of cirrhosis of the liver. Telangiectases on the other hand are deep blue blotches, often slightly raised.

*Dr. Johnson:* The spider naevus is a much brighter red.

*Dr. Barritt:* How often do marantic thrombi on the mitral valve give rise to carotid embolism?

*Professor Hewer:* I don't know that they do.

*Dr. Barritt:* I have seen one case of carcinomatosis with hemiplegia from a marantic thrombus.

*Professor Hewer:* I am surprised. They are usually firm, tough vegetations which do not break off and form emboli.

*Question:* Can we conclude that the best treatment of telangiectases is to leave them alone and merely treat the bleeding as it happens?

*Dr. Cates:* I do not think we have yet given Mr. Capper a fair trial to open the stomach and cauterize the telangiectases.

*Dr. Lloyd:* Is it really justifiable to open the stomach? Surely you find the lesions in the small intestine too?

*Dr. Cates:* I am not sure. Sometimes you do see cases in which, when you open them up, you find some localized areas of dilated vessels.

*Professor Neale:* I know a doctor who has this disease, he has a very big practice and lives a very busy life. The last time I saw him he was 66. Probably it is a good thing he didn't know about X-ray therapy.

*Dr. Cates:* Had our patient any arterio-venous aneurysms in the lungs?

*Dr. Johnson:* No.

## Figures and Tables

**Diagram I f1:**